# Racial and Ethnic Disparities in Hospitalization Outcomes Among Medicare Beneficiaries During the COVID-19 Pandemic

**DOI:** 10.1001/jamahealthforum.2021.4223

**Published:** 2021-12-23

**Authors:** Zirui Song, Xiaoran Zhang, Lindsey J. Patterson, C. Lowry Barnes, Derek A. Haas

**Affiliations:** 1 Department of Health Care Policy, Harvard Medical School, Boston, Massachusetts; 2 Department of Medicine, Massachusetts General Hospital, Boston, Massachusetts; 3 Avant-garde Health, Boston, Massachusetts; 4 University of Arkansas for Medical Sciences, Little Rock, Arkansas

## Abstract

**Importance:**

The COVID-19 pandemic has disproportionately affected racial and ethnic minority populations. However, racial and ethnic disparities in hospitalization outcomes during the pandemic—for both COVID-19 and non-COVID-19 hospitalizations—are poorly understood, especially among older populations.

**Objective:**

To assess racial and ethnic differences in hospitalization outcomes during the COVID-19 pandemic among Medicare beneficiaries.

**Design, Setting, and Participants:**

In the 100% traditional Medicare inpatient data, there were 31 771 054 unique beneficiaries in cross-section just before the pandemic (February 2020), among whom 26 225 623 were non-Hispanic White, 2 797 462 were Black, 692 994 were Hispanic, and 2 054 975 belonged to other racial and ethnic minority groups. There were 14 021 285 hospitalizations from January 2019 through February 2021, of which 11 353 581 were among non-Hispanic White beneficiaries, 1 656 856 among Black beneficiaries, 321 090 among Hispanic beneficiaries, and 689 758 among beneficiaries of other racial and ethnic minority groups. Sensitivity analyses tested expanded definitions of mortality and alternative model specifications.

**Exposures:**

Race and ethnicity in Medicare claims from the Social Security Administration.

**Main Outcomes and Measures:**

In-hospital mortality and mortality inclusive of discharges to hospice, deaths during 30-day readmissions, and 30-day all-cause mortality. Secondary outcomes included discharges to hospice and discharges to postacute care.

**Results:**

The decline in non–COVID-19 and emergence of COVID-19 hospitalizations were qualitatively similar among beneficiaries of different racial and ethnic minority groups through February 2021. In-hospital COVID-19 mortality was not significantly different among Black patients relative to White patients, but was 3.5 percentage points higher among Hispanic patients (95% CI, 2.9-4.1; *P* < .001) and other racial and ethnic minority patients relative to White counterparts (95% CI, 3.0-4.1; *P* < .001). For non–COVID-19 hospitalizations, in-hospital mortality among Black patients increased by 0.5 percentage points more than it increased among White patients (95% CI, 0.3-0.6; *P* < .001), a 17.5% differential increase relative to the prepandemic baseline. This gap was robust to expanded definitions of mortality. Hispanic patients had similar differential increases in expanded definitions of mortality and model specification. Disparities in discharges to hospice and postacute care were evident. In aggregate across COVID-19 and non–COVID-19 hospitalizations, mortality differentially increased among racial and ethnic minority populations during the pandemic.

**Conclusions and Relevance:**

In this cohort study, racial and ethnic disparities in mortality were evident among COVID-19 hospitalizations and widened among non–COVID-19 hospitalizations, motivating greater attention to health equity.

## Key Points

**Question:**

How did hospitalizations and racial and ethnic disparities in hospitalization outcomes change during the COVID-19 pandemic among patients with traditional Medicare?

**Findings:**

In this cohort study using 100% traditional Medicare inpatient data, comprising 31 771 054 beneficiaries and 14 021 285 hospitalizations from January 2019 through February 2021, the decline in non–COVID-19 and emergence of COVID-19 hospitalizations during the pandemic was qualitatively similar among beneficiaries of different racial and ethnic minority groups. In-hospital mortality for patients with COVID-19 was higher in racial and ethnic minority groups than in White patients, driven by a Hispanic-White gap; mortality among non–COVID-19 hospitalizations also differentially increased among patients in racial and ethnic minority groups relative to White patients, driven by an increased Black-White gap.

**Meaning:**

Racial and ethnic disparities in mortality were evident among COVID-19 hospitalizations and widened among non–COVID-19 hospitalizations among Medicare beneficiaries, motivating greater attention to health equity.

## Teaser

This cohort study examines hospitalizations and differential changes in mortality and related outcomes by race and ethnicity among Medicare beneficiaries during the COVID-19 pandemic.

## Introduction

The COVID-19 pandemic substantially disrupted US hospitals. Nationwide, hospitals experienced a surge of COVID-19 admissions, peaking in April 2020, that coincided with large declines in other domains of care. Although the decline in hospitalizations for acute conditions has been described,^1,2,3,4,5,6,7^ hospitalization outcomes during the pandemic remain poorly understood among both COVID-19 and non–COVID hospitalizations. This is especially true for older populations, who are clinically at higher risk of in-hospital mortality. In addition, racial and ethnic disparities in the case fatality of COVID-19 hospitalizations remain unclear, and such disparities among non–COVID-19 hospitalizations have been less explored.^8^ This dearth of data among non–COVID-19 hospitalizations is concerning, given the pandemic’s disproportionate burden on racial and ethnic minority groups and potential to exacerbate preexisting disparities in access and outcomes.^9,10,11,12,13^

Age-adjusted US death rates owing to COVID-19 among Black and Hispanic populations have been approximately double that among White populations.^14^ In many hospital cohort studies to date, however, Black and Hispanic patients with COVID-19 did not experience greater case fatality rates than their White counterparts after adjustment for clinical and social factors.^15,16,17,18,19,20,21^ However, because social determinants of health are correlated with race and ethnicity,^22,23^ adjustment for social factors likely attenuated or nullified the racial and ethnic disparities in mortality that these studies sought to measure. A recent meta-analysis of earlier studies, many of them from single institutions or smaller cohorts, found a lack of differences in in-hospital mortality between racial and ethnic minority groups and White patients adjusted for social factors, although it noted the limited generalizability of smaller-sample studies.^10^ To date, a nationwide examination of such racial and ethnic disparities in COVID-19 in-hospital mortality remains absent, notably among the elderly population, which is more clinically susceptible to COVID-19 mortality.

As hospitals responded to the COVID-19 pandemic, many faced a relatively fixed supply of beds and other physical and human resources in times of acute need, requiring internal redistribution of resources during the early pandemic months. Such redistribution, coupled with the simultaneous drop in demand for other hospital services, may have affected outcomes among non–COVID hospitalizations—which remained the dominant share of hospital admissions during the pandemic despite the drop off in utilization^8,24^—potentially widening racial and ethnic disparities in outcomes. These potential spillover effects of the pandemic have also not been fully examined in the nationwide Medicare population.

This study thus addresses 3 related gaps in evidence using the universe of Medicare fee-for-service hospitalizations from January 2019 through February 2021. First, how did hospitalization rates and in-hospital mortality change for Medicare beneficiaries of different racial and ethnic groups during the pandemic? Second, were there racial and ethnic disparities in inpatient COVID-19 case fatality rates among Medicare beneficiaries nationally, given the often lack of such disparities in previous smaller cohort studies? Finally, how did racial and ethnic disparities in hospitalization outcomes for non–COVID-19 patients change during the pandemic?

## Methods

### Patient Data and Variables

We used 100% Medicare fee-for-service hospital inpatient claims and enrollment data from the Centers for Medicare and Medicaid Services spanning January 2019 through February 2021. We measured the total number of hospitalizations, including COVID-19 and non–COVID-19 hospitalizations defined by the presence of a COVID-19 diagnosis at any point during the hospitalization. We used the *International Statistical Classification of Diseases and Related Health Problems, Clinical Modification, Tenth Revision (ICD-10) *code U07.1 to define COVID-19 hospitalizations.^25^ Hospitalizations included nonsurgical and surgical admissions. Outcomes included in-hospital mortality, defined as the discharge status of died/patient did not recover. We also assessed discharges to hospice and postacute care, the latter comprising inpatient rehabilitation and skilled nursing facilities. In sensitivity analyses, we expanded our mortality outcome to include deaths during readmissions within 30 days in the definition of in-hospital mortality as well as all-cause mortality within 30 days.

We assigned non–COVID-19 hospitalizations to clinical categories using major diagnostic category (MDC) and diagnosis-related group (DRG) definitions. These included 2 domains for non–COVID-19 intensive care: sepsis (DRGs 870-872) and admissions with ventilator support (DRGs 207-208). The additional non–COVID-19 domains comprised: neurologic (MDC 1), respiratory (MDC 4), cardiac (MDC 5), gastrointestinal (MDCs 6-7), orthopedic (MDC 8), kidney and genitourinary (MDCs 11-13), cancer (MDCs 16-17), and all other hospitalizations (eMethods 1 in the Supplement). This study followed the Strengthening the Reporting of Observational Studies in Epidemiology (STROBE) reporting guidelines and was approved by the institutional review board at Harvard University.^30^

### Race and Ethnicity

We defined race and ethnicity using the Medicare beneficiary race variable from the Social Security Administration. We compared Black, Hispanic, and other racial and ethnic minority group beneficiaries, respectively, to non-Hispanic White beneficiaries.^26,27^ Remaining racial and ethnic minority group members included Asian, North American Native, and beneficiaries of “unknown” or “other” race or ethnicity as reported by Medicare.

### Statistical Analysis

Analyses of COVID-19 hospitalizations comprised comparisons of mean outcomes between racial and ethnic groups, given that there were no such hospitalizations prepandemic. For our analyses of non–COVID-19 hospitalizations, we estimated an ordinary least squares difference-in-differences model at the hospital-month-race/ethnicity category level, allowing changes in outcomes attributable to race and ethnicity to vary by month, among racial and ethnic minority beneficiaries relative to their White counterparts. We defined the prepandemic period as January 2019 to February 2020 and pandemic period as April 2020 to February 2021. March 2020 was defined as the washout period, given geographic heterogeneity in the surge of cases that month and the introduction of the ICD-10-CM diagnosis code for COVID-19 in April 2020.

In our base specification, we adjusted for patient age, sex, share with end-stage kidney disease, and share with disability. We included hospital fixed effects, which accounted for time-invariant hospital characteristics, and month fixed effects (eMethods 2 in the Supplement).^28^ Standard errors were clustered by hospital. To assess clinical heterogeneity, we decomposed non–COVID-19 hospitalizations by their principal diagnostic category. Because rural or urban hospitals may receive a different selection of patients or specialize in different conditions, we also decomposed results by urban and rural location of hospitals using their core-based statistical area.^29^

We tested 3 expanded definitions of mortality in sensitivity analyses. Because in-hospital mortality could be substituted by discharges to hospice, we first included discharges to hospice within mortality. Second, we redefined mortality to include deaths during 30-day readmissions after the index hospitalization. Third, we examined all-cause mortality within 30 days of the index admission. Finally, we tested for differences in admissions from skilled nursing facilities, which may help explain differences in discharges to postacute care.

Additional sensitivity analyses tested the robustness of results to changes in the model, including adjustment for core-based statistical area COVID-19 infection rates as a measure of the intensity of the pandemic, including hospital random effects rather than fixed effects, and restricting the sample to beneficiaries aged 65 years and older. Falsification tests varied the timing of the pandemic.

In secondary descriptive analyses, we explored potential differential risk selection associated with the pandemic by examining the characteristics of beneficiaries admitted before and during the COVID-19 pandemic. This enabled us to calculate the patient characteristics of non–COVID-19 admissions that would have been expected to occur in the absence of the pandemic, which may help explain differential changes in non–COVID-19 in-hospital mortality by race and ethnicity. We also directly tested for differential changes in risk with the Centers for Medicare and Medicaid Services Hierarchical Condition Category (CMS-HCC) risk score, calculated using age, sex, and inpatient clinical diagnoses, as the dependent variable.

Two-sided *P* values were calculated for the main outcomes; we reported 95% CIs for other comparisons. Analyses were conducted using Stata statistical software, version 16, from March 23, 2021, to October 25, 2021.

## Results

### Changes in Hospitalizations

The racial and ethnic composition of COVID-19 and non–COVID-19 Medicare inpatients is shown in eTable 1 in the Supplement. Beneficiaries hospitalized with COVID-19 were more likely to be from racial and ethnic minority groups relative to hospitalized beneficiaries prepandemic. Table 1 provides the volume, rates, and characteristics of hospitalized beneficiaries before and after the COVID-19 pandemic for each racial and ethnic category. Relative to White patients with COVID-19, those of racial and ethnic minority groups were younger, similar in inpatient risk score, and more likely to have end-stage kidney disease, a disability, and be dually eligible for Medicaid (Table 1).

**Table 1.** Characteristics of COVID-19 and Non–COVID-19 Hospitalizations in Medicare^a^

Characteristic	Medicare patients, %		
	Pre–COVID-19	During COVID-19	
		Non–COVID-19	COVID-19
**Black**			
Hospitalizations			
Per month, No.	69 364	49 432	7619
Rate^b^	23.6	18.2	2.8
Age, y	65.7	65.5	68.9
Sex (% female)	55.2	53.8	53.1
Risk score^c^	2.0	2.1	2.2
ESKD	17.1	18.0	15.0
Disability	30.0	29.3	21.8
Dual eligible	51.4	51.7	55.7
**Hispanic**			
Hospitalizations			
Per month, No.	13 234	8896	2440
Rate^b^	18.6	12.9	3.6
Age, y	64.3	63.1	68.0
Sex (% female)	52.0	50.1	46.2
Risk score^c^	1.9	2.0	2.0
ESKD	19.0	20.3	16.3
Disability	32.8	34.6	23.3
Dual eligible	71.8	70.8	64.9
**White**			
Hospitalizations			
Per month, No.	479 560	350 164	37 080
Rate^b^	17.9	13.4	1.4
Age, y	73.1	73.0	74.6
Sex (% female)	53.9	53.0	49.3
Risk score^c^	1.9	2.0	2.1
ESKD	3.6	3.7	3.3
Disability	12.5	12.1	8.6
Dual eligible	21.4	20.8	27.3
**Other racial and ethnic minority groups**			
Hospitalizations			
Per month, No.	28 207	21 206	3430
Rate^b^	13.7	10.1	1.6
Age, y	69.5	68.7	70.8
Sex (% female)	47.7	46.6	45.4
Risk score^c^	1.9	2.0	2.0
ESKD	10.9	11.3	9.9
Disability	15.3	16.0	12.0
Dual eligible	43.1	41.1	47.8

Abbreviations: COVID-19, coronavirus disease 2019; ESKD, end-stage kidney disease.

^a^ Data are from the Centers for Medicare and Medicaid Services 100% inpatient claims files, which capture all Medicare fee-for-service hospitalizations. Pre–COVID-19 denotes January 2019 through February 2020; during COVID-19 denotes April 2020 through February 2021. Other racial and ethnic minority group members included Asian, North American Native, and beneficiaries of “unknown” or “other” race as reported by the Medicare data.

^b^ Hospitalizations per 1000 beneficiaries per month.

^c^ The risk score was calculated using the Centers for Medicare and Medicaid Services Hierarchical Condition Category (CMS-HCC) model with the beneficiaries’ age, sex, and inpatient clinical diagnoses.

**Figure 1.**
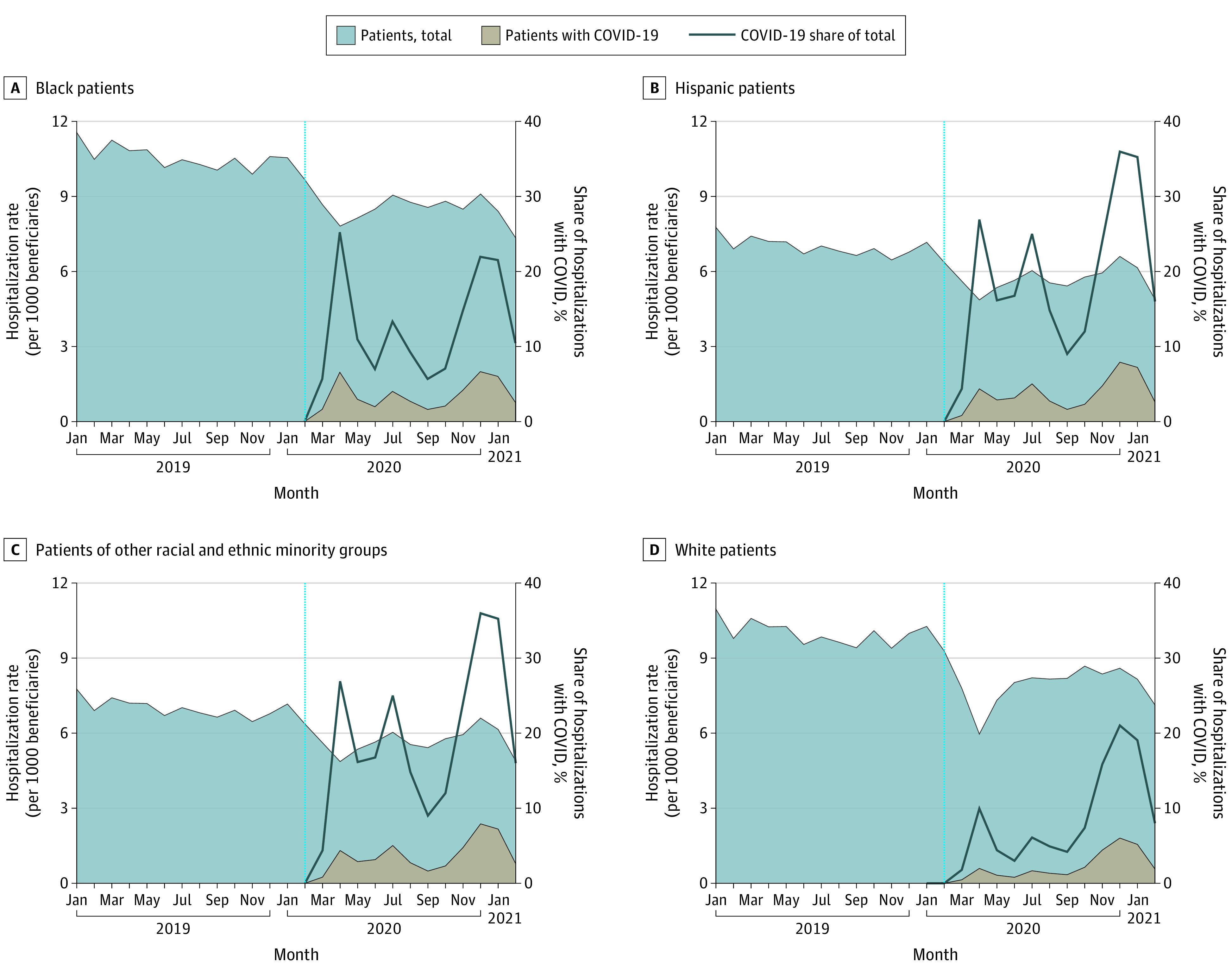
Total Medicare Hospitalizations and Share of Hospitalizations for Patients With COVID-19^a^ ^a^The rate of COVID-19 and non–COVID-19 hospitalizations for Black, Hispanic, and other racial and ethnic minority groups, and non-Hispanic White traditional Medicare beneficiaries are shown in the shaded regions (left vertical axis). The share of total hospitalizations representing COVID-19 hospitalizations is represented by the line plot (right vertical axis). Other racial and ethnic minority group members included Asian, North American Native, and beneficiaries of “unknown” or “other” race as reported by the Medicare beneficiary race variable from the Social Security Administration. COVID-19 hospitalizations comprise patients who had a diagnosis of COVID-19 during the hospitalization, whether or not COVID-19 was the chief reason for hospitalization. The vertical line denotes February 2020, the last prepandemic month.

White beneficiaries experienced a decline in non–COVID-19 hospitalizations from 17.9 per 1000 beneficiaries per month prepandemic to an average of 13.4 per 1000 beneficiaries per month through February 2021 (25.0% decline), whereas 1.4 COVID-19 hospitalizations per 1000 beneficiaries per month emerged. Meanwhile, Black, Hispanic, and other racial and ethnic minority beneficiaries saw reductions in non–COVID-19 hospitalizations of 22.9%, 30.6%, and 26.4%, respectively. Black and Hispanic beneficiaries were hospitalized for COVID-19 at rates of 2.8 and 3.6 per 1000 beneficiaries per month, respectively (Table 1, Figure 1). Variations by clinical domain are shown in eFigure 1 and eTable 2 in the Supplement. Urban and rural hospitals generally experienced qualitatively similar declines in non–COVID-19 hospitalizations (eTable 3 in the Supplement).

### Disparities in COVID-19 In-Hospital Mortality

Relative to White patients with COVID-19, those of racial and ethnic minority groups were younger, similar in their inpatient risk scores, and more likely to have end-stage kidney disease, a disability, and be dually eligible for Medicaid through comparisons of unadjusted means (Table 1). Unadjusted in-hospital mortality among COVID-19 hospitalizations was 16.6% among White patients, 17.0% among Black patients, 21.7% among Hispanic patients, and 21.0% among other racial and ethnic minority group patients (Table 2).

**Table 2.** Changes in Medicare Hospitalization Outcomes, Racial and Ethnic Minority Groups vs White Patients^a^

Variable	Medicare patients, %								Between-group difference^c^											
	White		Black		Hispanic		Other racial and ethnic minority groups^b^		Black vs White				Hispanic vs White				Other racial and ethnic minority groups^b^ vs White			
	Pre-COVID	During COVID	Pre-COVID	During COVID	Pre-COVID	During COVID	Pre-COVID	During COVID	Unadjusted, %	Adjusted, % (95% CI)	Change, %	*P* value	Unadjusted, %	Adjusted, % (95% CI)	Change, %	*P* value	Unadjusted, %	Adjusted, % (95% CI)	Change, %	*P* value
**COVID-19 hospitalizations^d^**																				
In-hospital mortality^e^	NA	16.63	NA	16.97	NA	21.72	NA	20.97	0.34	0.11 (−0.24 to 0.47)	NA	.53	5.09	3.49 (2.90 to 4.08)	NA	<.001	4.34	3.5 (2.95 to 4.06)	NA	<.001
Discharge to hospice	NA	5.78	NA	3.76	NA	3.70	NA	3.30	2.02	−1.20 (−1.40 to −1.00)	NA	<.001	−2.08	−0.6 (0.91 to −0.29)	NA	<.001	−2.47	−1.03 (−1.29 to −0.78)	NA	<.001
Discharge to post-acute care	NA	22.94	NA	24.20	NA	14.47	NA	16.74	1.25	1.56 (1.13 to 1.99)	NA	<.001	−8.48	−9.34 (−9.91 to −8.77)	NA	<.001	−6.20	−5.76 (−6.25 to −5.26)	NA	<.001
**Non–COVID-19 hospitalizations**																				
In-hospital mortality	2.80	3.30	2.75	3.55	2.77	3.58	3.32	3.79	0.30	0.48 (0.33 to 0.63)	17.5	<.001	0.30	0.29 (−0.04 to 0.62)	10.6	.08	−0.03	0.07 (−0.17 to 0.30)	2.1	0.57
Discharge to hospice	3.19	3.91	2.55	3.24	2.54	3.24	2.47	3.21	−0.04	0.05 (−0.09 to 0.19)	1.9	.50	−0.03	0.19 (−0.11 to 0.50)	7.6	.21	0.01	0.14 (−0.07 to 0.36)	5.8	0.19
Discharge to postacute care	24.11	20.48	21.90	18.89	17.95	14.47	19.44	15.47	0.61	0.29 (−0.07 to 0.66)	1.3	.11	0.15	0.32 (−0.41 to 1.05)	1.8	.39	−0.35	0.47 (−0.02 to 0.96)	2.4	.06
**All traditional hospitalizations**																				
In-hospital mortality	2.80	4.58	2.75	5.34	2.77	7.48	3.32	6.19	0.81	0.95 (0.79 to 1.11)	34.5	<.001	2.93	2.86 (2.49 to 3.24)	103.5	<.001	1.08	1.14 (0.85 to 1.43)	34.4	<.001

Abbreviations: COVID-19, coronavirus disease 2019; NA, not applicable.

^a^ In-hospital mortality reflects a discharge status of death. Pre-COVID denotes January 2019 through February 2020; during COVID-19 denotes April 2020 through February 2021.

^b^ Other racial and ethnic minority group members included Asian, North American Native, and beneficiaries of “unknown” or “other” race as reported by the Medicare beneficiary race variable from the Social Security Administration.

^c^ Unadjusted between-group differences were calculated as the difference in the changes between the racial and ethnic minority groups and White categories. Adjusted between-group differences were estimates from the statistical model, adjusted for age, sex, disability, end-stage kidney disease, month fixed effects, and hospital fixed effects, with standard errors clustered at the hospital level.

^d^ Given there were no COVID-19 hospitalizations pre–COVID-19, the between-group difference was a cross-sectional comparison between racial and ethnic minority groups and white beneficiaries using data during the pandemic.

^e^ In-hospital mortality reflects a discharge status of death.

In adjusted analyses, in-hospital mortality was not significantly different among Black patients with COVID-19 relative to White patients, but was 3.5 percentage points higher among both Hispanic patients relative to White patients (95% CI, 2.9-4.1; *P* < .001) and patients in other racial and ethnic minority groups relative to their White counterparts (95% CI, 3.0-4.1; *P* < .001) (Table 2 and eTable 4 in the Supplement). These latter disparities were robust to expanded definitions of mortality (eTable 4 in the Supplement), and results were similar in rural and urban hospitals (eTable 5 in the Supplement).

Relative to White patients with COVID-19, Black, Hispanic, and remaining racial and ethnic minority patients with COVID-19 appeared less likely to be discharged to hospice (Table 2). Hispanic and other racial and ethnic minority patients with COVID-19 were similarly discharged less frequently to postacute care relative to White patients (Table 2), consistent with their lower rates of admission from skilled nursing facilities (eTable 4 in the Supplement). In contrast, Black patients were more likely discharged to postacute care, and more likely to have been admitted from skilled nursing facilities, relative to their White counterparts (Table 2). Among patients with COVID-19 discharged to postacute care, White patients were less likely to have end-stage kidney disease, disability, and dual eligibility than any category of racial and ethnic minority patients (eTable 6 in the Supplement).

### Disparities in Non–COVID-19 In-Hospital Mortality

**Figure 2.**
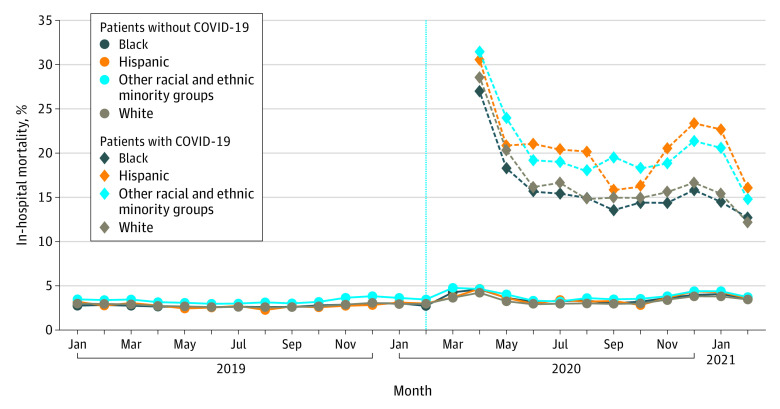
In-Hospital Mortality Among COVID-19 and All Other Hospitalizations^a^ ^a^In-hospital mortality reflects a discharge status of death and is shown among COVID-19 and non-COVID hospitalizations for Black, Hispanic, other racial and ethnic minority groups, and non-Hispanic White beneficiaries. Other racial and ethnic minority group members included Asian, North American Native, and beneficiaries of “unknown” or “other” race as reported by the Medicare beneficiary race variable from the Social Security Administration. The COVID-19 category comprises all hospitalizations of patients who had a diagnosis of COVID-19, whether or not COVID-19 was the chief reason for hospitalization. The vertical line denotes February 2020, the last prepandemic month.

During the pandemic, non–COVID-19 hospitalizations remained the large majority of Medicare hospitalizations among all racial and ethnic groups (Table 1). Unadjusted in-hospital mortality for non–COVID-19 hospitalizations increased across racial and ethnic groups relative to prepandemic (Figure 2), with variation across clinical categories (eFigure 2 in the Supplement).

In adjusted analyses, in-hospital mortality among non–COVID-19 Black patients increased by 0.48 percentage points more than it increased among non–COVID-19 White patients (95% CI, 0.33-0.63; *P* < .001)—representing a 17.5% increase over the prepandemic mortality rate among Black inpatients (Table 2). This was explained by patients admitted for sepsis, neurologic, cardiac, gastrointestinal, and kidney and genitourinary indications (Table 3). This widened Black-White mortality gap was robust to expanded definitions of mortality (eTable 4 in the Supplement) and was similar across rural and urban hospitals (eTable 5 in the Supplement). Hispanic non–COVID-19 patients did not exhibit differential changes in in-hospital mortality relative to their White counterparts (Table 2), but did experience similar differential increases in mortality under expanded definitions that included hospice, deaths during 30-day readmissions, and 30-day all-cause mortality (eTable 4 in the Supplement). No differential changes in mortality were found among patients in other racial and ethnic minority groups.

**Table 3.** Changes in Medicare Non-COVID-19 In-Hospital Mortality, Racial and Ethnic Minority Groups vs White Patients^a^

Non–COVID-19 hospitalizations	%								Between-group difference^c^											
	White		Black		Hispanic		All racial and ethnic minority groups^b^		Black vs White				Hispanic vs White				All racial and ethnic minority groups vs White			
	Pre-COVID	During COVID	Pre-COVID	During COVID	Pre-COVID	During COVID	Pre-COVID	During COVID	Unadjusted, %	Adjusted, % (95% CI)	Change, %	*P* value	Unadjusted, %	Adjusted, % (95% CI)	Change, %	*P* value	Unadjusted, %	Adjusted, % (95% CI)	Change, %	*P* value
In-hospital mortality																				
Sepsis	8.53	10.62	9.80	12.76	8.01	11.55	9.41	11.70	0.88	1.50 (0.64 to 2.36)	15.3	<.001	1.45	1.57 (0.09 to 3.06)	19.7	.04	0.20	0.11 (−1.11 to 1.33)	1.2	.86
Ventilators	26.43	30.84	22.75	28.57	26.49	31.62	29.31	32.90	1.40	3.42 (−0.46 to 7.29)	15.0	.08	0.71	−0.74 (−10.17 to 8.68)	−2.8	.88	−0.83	0.49 (−6.07 to 7.04)	1.7	.88
Neurologic	4.04	4.28	3.12	3.83	3.75	4.09	5.05	5.25	0.47	0.58 (0.06 to 1.11)	18.7	.03	0.10	−0.81 (−2.16 to 0.54)	−21.6	.24	−0.05	0.22 (−0.74 to 1.18)	4.3	.66
Respiratory	2.00	2.72	1.42	2.09	1.84	2.96	2.33	2.95	−0.05	−0.09 (−0.40 to 0.23)	−6.1	.59	0.40	0.40 (−0.43 to 1.23)	21.8	.35	−0.10	0.01 (−0.54 to 0.57)	0.5	.97
Cardiac	2.59	2.89	2.23	2.67	2.47	2.84	2.88	3.17	0.14	0.31 (0.02 to 0.60)	13.9	.04	0.07	0.26 (−0.41 to 0.93)	10.5	.45	−0.02	−0.08 (−0.57 to 0.40)	−2.9	.73
Gastrointestinal	1.88	2.09	1.96	2.33	1.62	2.10	2.07	2.13	0.15	0.66 (0.34 to 0.98)	33.7	<.001	0.27	0.40 (−0.28 to 1.08)	24.7	.25	−0.15	−0.03 (−0.58 to 0.52)	−1.4	.92
Orthopedic^d^	0.62	0.78	0.65	0.80	NA	NA	0.57	0.75	−0.02	0.01 (−0.25 to 0.27)	1.5	.94	NA	0.50 (0.11 to 0.90)	NA	.01	0.01	−0.06 (−0.38 to 0.25)	−10.8	.70
Kidney and Genitourinary^d^	1.35	1.51	1.22	1.64	1.05	NA	1.21	1.30	0.26	0.41 (0.09 to 0.73)	33.5	.01	NA	−0.11 (−0.80 to 0.58)	−10.8	.75	−0.07	−0.02 (−0.55 to 0.50)	−2.0	.93
Cancer^d^	3.15	3.14	1.91	2.25	NA	NA	3.15	3.36	0.35	0.7 (−0.05 to 1.46)	36.8	.07	NA	0.87 (−1.16 to 2.90)	NA	.40	0.21	0.71 (−0.74 to 2.16)	22.6	.34
Other	2.49	2.82	2.19	2.74	1.89	2.45	2.68	2.90	0.21	0.28 (−0.05 to 0.61)	12.6	.10	0.23	0.13 (−0.46 to 0.72)	7.0	.66	−0.12	−0.08 (−0.64 to 0.49)	−2.8	.79

Abbreviations: COVID-19, coronavirus disease 2019; NA, not applicable.

^a^ In-hospital mortality reflects a discharge status of death. Pre–COVID-19 denotes January 2019 through February 2020; during COVID-19 denotes April 2020 through February 2021.

^b^ Remaining racial and ethnic minority group members included Asian, North American Native, and beneficiaries of “unknown” or “other” race as reported by the Medicare beneficiary race variable from the Social Security Administration.

^c^ Unadjusted between-group differences were calculated as the difference in the changes between the racial and ethnic minority groups and White categories. Adjusted between-group differences were estimates from the statistical model, adjusted for age, sex, disability, end-stage kidney disease, month fixed effects, and hospital fixed effects, with standard errors clustered at the hospital level.

^d^ Mortality rates for cells containing 10 or fewer deaths were suppressed to protect confidentiality.

Based on the decline in non–COVID-19 hospitalizations during the pandemic, the volume and patient characteristics of non–COVID-19 admissions that would have been expected to occur in the absence of the pandemic are shown in eTable 7 in the Supplement. In our difference-in-differences model, the average inpatient risk score differentially increased by 0.02 for Black, Hispanic, and other minority inpatients relative to their White counterparts (eTable 8 in the Supplement). To the extent this reflects actual risk rather than coding behavior or measurement error from inpatient claims alone, this could be consistent with non–COVID-19 White beneficiaries who stayed home during the pandemic being of differentially higher risk, leading to differentially higher-risk racial and ethnic minority patients admitted to hospitals. This differentially increased risk among Black inpatients may help explain the widening non–COVID-19 mortality gap relative to their White counterparts.

### Aggregate In-Hospital Mortality

Combined with COVID-19 hospitalizations, aggregate in-hospital mortality increased 0.95 percentage points more among Black patients relative to White patients (95% CI, 0.79-1.11; *P* < .001) or a 34.5% increase from baseline, 2.86 percentage points more among Hispanic patients relative to White (95% CI, 2.49-3.24; *P* < .001) or a 103.5% increase from baseline, and 1.14 percentage points more among remaining racial and ethnic minority patients relative to White patients (95% CI, 0.85-1.43; *P* < .001) or a 34.4% increase from baseline (Table 2).

In sensitivity analyses, our main estimates were qualitatively similar after changes in the statistical model and covariates. Falsification tests supported the timing of the initial COVID-19 surge in April 2020 and that month as the start of the treatment period (eTables 9-11 in the Supplement).

## Discussion

Using complete traditional Medicare inpatient data, we found 3 key changes in hospital care associated with the COVID-19 pandemic through 2020. First were the changes in hospitalization volume. Non–COVID-19 hospitalizations declined substantially among both racial and ethnic minority and White beneficiaries, which adds to earlier evidence of declines in health care use.^1,2,3,4,5,6,7,8^ The average rate of Black and Hispanic COVID-19 hospitalizations exceeded that of White beneficiaries through February 2021.

Second, COVID-19 in-hospital mortality was higher among Hispanic and other racial and ethnic minority patients than among White patients. These disparities were robust to sensitivity analyses and expanded definitions of mortality, including all-cause mortality within 30 days and defining discharges to hospice within mortality. Black patients did not experience significant differences in COVID-19 in-hospital mortality relative to their White counterparts, although had lower 30-day all-cause mortality and when hospice was included within mortality. Importantly, we did not adjust for social determinants of health, such as income and education, because they are correlated with race and ethnicity. Such adjustment would attenuate the differences in outcomes associated with race and ethnicity that we aimed to measure. Our findings contrast with several prior studies that did adjust for social factors, which often found no racial or ethnic differences in COVID-19 hospital case fatality in smaller cohorts.^10^ To the extent that adjustment for social factors attenuates these disparities, it suggests that societal or structural factors, such as structural racism, could affect hospitalization outcomes. The 100% traditional Medicare inpatient data through February 2021 was also a notable difference relative to earlier studies. Our study builds on prior work that found Black-White Medicare disparities in 30-day inpatient mortality or discharge to hospice after an initial COVID-19 diagnosis.^31^

Hispanic and other racial and ethnic minority patients with COVID-19 were discharged to postacute facilities, and admitted from skilled nursing facilities, less than White patients with COVID-19. In contrast, Black patients with COVID-19 were more likely than their White counterparts to be discharged to postacute care and admitted from skilled nursing facilities. To the extent that postacute, rehabilitative, or nursing resources improve recovery from disease,^32,33^ it may help explain the differences in all-cause 30-day mortality noted above.

Finally, we uncovered disparities in non–COVID-19 in-hospital mortality during the pandemic. As non–COVID-19 admissions declined in Medicare, Black inpatients experienced a nearly 0.5 percentage-point (17.5%) differential increase in in-hospital mortality compared with White inpatients, robust to expanded definitions of mortality. Hispanic inpatients similarly exhibited differential increases in expanded definitions of mortality, including 30-day all-cause mortality. These results add to an earlier study using data from a large physician group across about 200 hospitals that found an analogous spillover effect during April 2020, though it was statistically insignificant thereafter.^3^ Our results suggest that this widening disparity persisted for many beneficiaries, especially Black beneficiaries.

Explanations for this persistently widened disparity in non–COVID-19 mortality could include differential access to COVID-19 testing or recording of test results, changes in case mix, quality of care, and other societal inputs, such as access to care, personal resources, and social determinants of health. We found suggestive evidence of changes in case mix because differentially higher-risk White beneficiaries appeared to have avoided inpatient care. This could arise from demand-side factors (eg, higher-risk White patients may have stayed home more or had more access to outpatient or telehealth that may substitute for inpatient care) or supply-side factors (eg, changes in hospital capacity or admission patterns leading to higher-risk racial and ethnic minority patients or lower-risk White patients hospitalized). Changes in quality of care may have contributed if, for example, clinician attention or other resources were diverted away from racial and ethnic minority non–COVID-19 patients during the pandemic. Broader societal inequities, such as structural racism, may contribute to such explanations.

### Limitations

We note several limitations. Our base definition of in-hospital mortality was narrow and included only the discharge status of death. We could not measure differential changes in access to hospitals. If racial and ethnic minority beneficiaries had disproportionate challenges getting admitted into hospitals during the pandemic, they may have had increased deaths out of hospital that were not reflected in the base definition. However, our broader definitions of mortality including discharges to hospice, mortality during 30-day readmissions, and 30-day all-cause mortality (2 specifications) did not generally change the findings qualitatively.

Second, surges of COVID-19 cases were geographically and temporally diverse, and the ICD-10 code for COVID-19 was not implemented until April 2020, potentially leading to under-coding of COVID-19 in earlier admissions.^34,35^ To help alleviate these concerns, we abstracted to the month level and considered March 2020 a washout period. Results were also robust to the inclusion of area-level time-varying COVID-19 infection rates. Finally, although we adjusted for time-invariant hospital attributes, time-varying factors may have confounded our estimates because hospitals reallocated physical or human resources in response to the pandemic. Our results were similar in the random effects model.

More broadly, such investigations using claims data are not able to adjust for severity of COVID-19, which can present in clinically heterogeneous ways. Therefore, small differences in clinical thresholds or patterns of admission may lead to large differences in mortality measures.

Finally, because COVID-19 affected the health care system and society in many ways, the mechanisms of the pandemic’s effect on hospital care are difficult to isolate. The pandemic may have affected supply side inputs, including hospital resources (eg, staff and equipment) and clinician behavior (clinical decision-making, attention, and other dimensions of quality), and demand side factors such as care-seeking behavior.

## Conclusions

This cohort study provides novel evidence on hospitalizations and outcomes in Medicare beneficiaries during the COVID-19 pandemic. Among COVID-19 and non–COVID-19 hospitalizations, racial and ethnic disparities in mortality were evident. As the pandemic evolves, efforts to understand the sources of pandemic-associated disparities and to improve health equity are needed.

## Notes

Supplement. eMethods 1. Categories of Hospitalizations eMethods 2. Empirical Model Specification eFigure 1. Hospitalization Rate by Clinical Category eFigure 2. In-Hospital Mortality by Clinical Category eTable 1. Racial and Ethnic Composition of COVID-19 and Non-COVID-19 Hospitalizations in Medicare eTable 2. Changes in Monthly Hospitalizations by Racial and Ethnic Group and Clinical Category eTable 3. Changes in Monthly Hospitalizations by Racial and Ethnic Group and Hospital Location eTable 4. In-Hospital Mortality, Black vs White, Hispanic vs White, and Remaining Racial and Ethnic Minority Groups vs White eTable 5. Differential Changes in Hospitalization Outcomes eTable 6. Characteristics of Medicare Hospitalizations by Discharge Status eTable 7. Absent Non-COVID-19 Inpatients During the Pandemic eTable 8. Differential Changes in Risk Score eTable 9. Sensitivity Analyses and Falsification Test, Black vs White eTable 10. Sensitivity Analyses and Falsification Test, Hispanic vs. White eTable 11. Sensitivity Analyses and Falsification Test, Remaining Racial and Ethnic Minority Groups vs. White
