# Supplementary material for: Racial and Ethnic Disparities in Hospitalization Outcomes Among Medicare Beneficiaries During the COVID-19 Pandemic
Source: JAMA Health Forum. 2021 Dec 23;2(12):e214223. doi: 10.1001/jamahealthforum.2021.4223 (PMC8796953; doi:10.1001/jamahealthforum.2021.4223)
Supplement: Supplement. — eMethods 1. Categories of Hospitalizations eMethods 2. Empirical Model Specification eFigure 1. Hospitalization Rate by Clinical Category eFigure 2. In-Hospital Mortality by Clinical Category eTable 1. Racial and Ethnic Composition of COVID-19 and Non-COVID-19 Hospitalizations in Medicare eTable 2. Changes in Monthly Hospitalizations by Racial and Ethnic Group and Clinical Category eTable 3. Changes in Monthly Hospitalizations by Racial and Ethnic Group and Hospital Location eTable 4. In-Hospital Mortality, Black vs White, Hispanic vs White, and Remaining Racial and Ethnic Minority Groups vs White eTable 5. Differential Changes in Hospitalization Outcomes eTable 6. Characteristics of Medicare Hospitalizations by Discharge Status eTable 7. Absent Non-COVID-19 Inpatients During the Pandemic eTable 8. Differential Changes in Risk Score eTable 9. Sensitivity Analyses and Falsification Test, Black vs White eTable 10. Sensitivity Analyses and Falsification Test, Hispanic vs. White eTable 11. Sensitivity Analyses and Falsification Test, Remaining Racial and Ethnic Minority Groups vs. White [file jamahealthforum-e214223-s001.pdf]

Supplemental Online Content

Song Z, Zhang X, Patterson LJ, et al. Racial and ethnic disparities in hospitalization outcomes among Medicare beneficiaries during the COVID-19 pandemic. *JAMA Health Forum*. Published online December 23, 2021. doi:10.1001/jamahealthforum.2021.4223

eMethods 1. Categories of Hospitalizations

eMethods 2. Empirical Model Specification

eFigure 1. Volume of Hospitalizations by Clinical Category

eFigure 2. In-Hospital Mortality by Clinical Category

eTable 1. Race/Ethnicity of Covid-19 and Non-COVID-19 Hospitalizations in Medicare

eTable 2. Changes in Monthly Hospitalizations by Race/Ethnicity and Clinical Category

eTable 3. Changes in Monthly Hospitalizations by Race/Ethnicity and Hospital Location

eTable 4. In-Hospital Mortality, Black vs. White and Hispanic vs. White

eTable 5. Differential Changes in Hospitalization Outcomes

eTable 6. Characteristics of Medicare Hospitalizations by Discharge Status

eTable 7. Absent Non-COVID-19 Inpatients During the Pandemic

eTable 8. Differential Changes in Risk Score

eTable 9. Sensitivity Analyses and Falsification Test

This supplemental material has been provided by the authors to give readers additional information about their work.

© 2021 Song Z et al. *JAMA Health Forum.*

eMethods 1. Categories of Hospitalizations[1]

	ICD-10 Diagnosis Codes	Major Diagnostic Categories (MDCs)	Diagnosis-Related Groups (DRGs)
COVID-19 Hospitalizations	U07.1	All	All
Non-COVID-19 Hospitalizations	All except U07.1	All	All
Sepsis	All except U07.1	--	870-872
Ventilators	All except U07.1	--	207-208
Neurologic	All except U07.1	1	020-103
Respiratory	All except U07.1	4	163-207
Cardiac	All except U07.1	5	215-316
Gastrointestinal	All except U07.1	6-7	326-446
Orthopedic	All except U07.1	8	453-566
Renal/genitourinary	All except U07.1	11-13	652-761
Cancer	All except U07.1	16-17	799-849
Other	All except U07.1	All other	All except 870-872

[1] Major Diagnostic Categories (MDCs) map to diagnosis-related groups (DRGs).

© 2021 Song Z et al. *JAMA Health Forum.*

eMethods 2. Empirical Model Specification

Admission-level outcomes:

For admission-level outcomes (e.g., in-hospital mortality, length of stay), we estimated the following event study model:

$$y_{iht} = \beta_0 + \sum_{t=1/19}^{t=12/20} \beta_t(R_i \times \mathbf{1}(month_t)) + \Gamma X_i + \theta_h + \delta_t + \varepsilon_{iht}$$

where y_{iht} is the outcome of interest for admission *i* to hospital *h* in month *t*; R_i indicates whether the admission is for a patient of a racial/ethnic minority group and is interacted with a full set of month/year fixed effects; X_i is vector of admission-level characteristics (age category, sex, race category, disability, and end-stage renal disease [ESRD]); θ_h are hospital fixed effects; δ_t are month fixed effects; and standard errors are clustered at the hospital level. The coefficients of interest are β_t estimates, which are presented relative to February 2020.

To estimate the average effect in the post-COVID-19 period, we constrain the monthly April 2020-December 2020 coefficients, but allow the effect of race to vary over all other months:

$$y_{iht} = \beta_0 + \sum_{t=1/19}^{t=3/20} \beta_t(R_i \times \mathbf{1}(month_t)) + \beta_{post}(R_i \times post_t) + \Gamma X_i + \theta_h + \delta_t + \varepsilon_{iht}$$

Hospital-level outcomes:

For hospital-level outcomes (e.g., volume of hospitalizations, Medicare spending), we used similar model specifications:

April specific effect:

$$y_{jht} = \beta_0 + \sum_{t=1/19}^{t=9/20} \beta_t(R_j \times \mathbf{1}(month_t)) + \Gamma X_j + \theta_h + \delta_t + \varepsilon_{jht}$$

where y_{jht} is the outcome of interest for race category *j* to hospital *h* in month *t*; R_j indicates whether the race category is a racial/ethnic minority group; and X_j is a vector of time-vary characteristics (share of admissions female, share of admissions in each age category, share of admissions with a disability, share of admissions with ESRD).

April-September average month effect:

$$y_{jht} = \beta_0 + \sum_{t=1/19}^{t=3/20} \beta_t(R_j \times \mathbf{1}(month_t)) + \beta_{post}(R_j \times post_t) + \Gamma X_j + \theta_h + \delta_t + \varepsilon_{jht}$$

© 2021 Song Z et al. *JAMA Health Forum.*

eFigure 1. Hospitalization Rate by Clinical Category

The COVID and non-COVID hospitalization rate (per 1,000 Medicare beneficiaries) for White patients (gray diamonds), Black patients (green circles), Hispanic patients (purple circles), and patients of remaining racial and ethnic minority groups (orange circles). Remaining racial and ethnic minority group members included Asian, North American Native, and beneficiaries of “unknown” or “other” race as reported by the Medicare beneficiary race variable from the Social Security Administration. COVID hospitalizations comprise patients who had a diagnosis of COVID-19 during the hospitalization, whether or not COVID-19 was the chief reason for hospitalization. The vertical line denotes February 2020, the last pre-pandemic month.

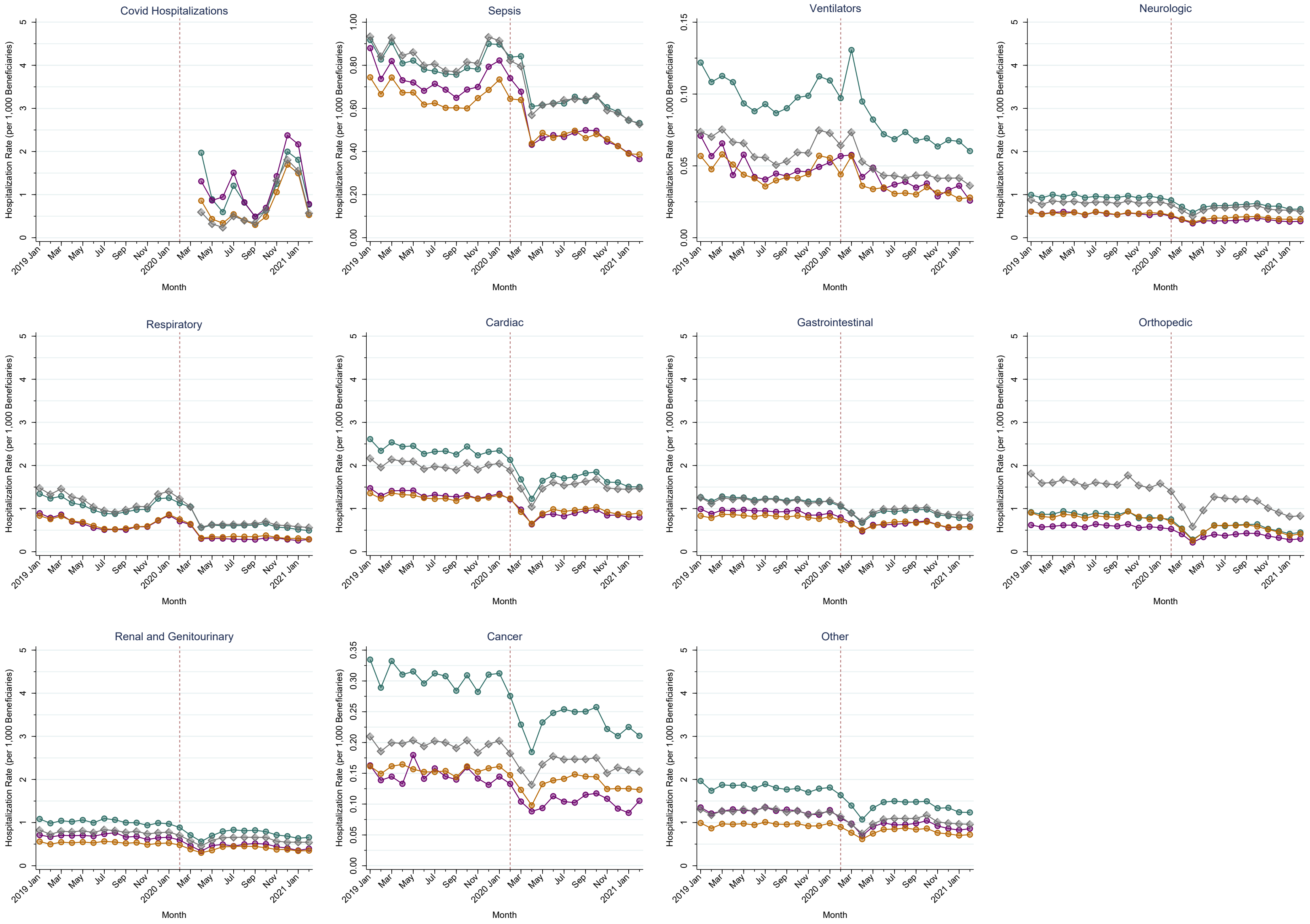

© 2021 Song Z et al. *JAMA Health Forum.*

eFigure 2. In-hospital Mortality by Clinical Category

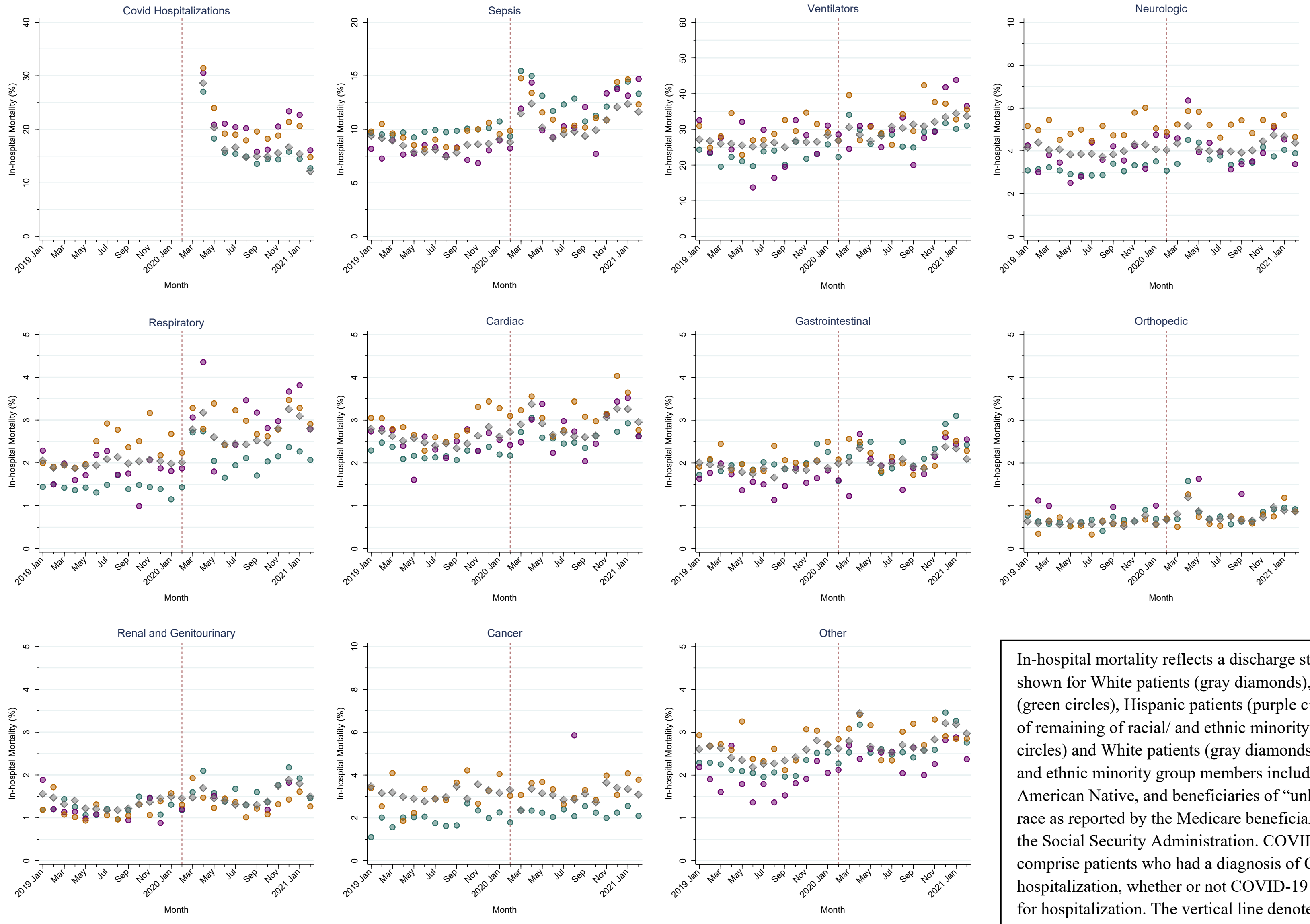

In-hospital mortality reflects a discharge status of death and is shown for White patients (gray diamonds), Black patients (green circles), Hispanic patients (purple circles), and patients of remaining of racial/ and ethnic minority groups (green orange circles) and White patients (gray diamonds). Remaining racial and ethnic minority group members included Asian, North American Native, and beneficiaries of "unknown" or "other" race as reported by the Medicare beneficiary race variable from the Social Security Administration. COVID hospitalizations comprise patients who had a diagnosis of COVID-19 during the hospitalization, whether or not COVID-19 was the chief reason for hospitalization. The vertical line denotes February 2020, the last pre-pandemic month. Mortality rates for cells containing 10 or fewer deaths were suppressed to protect confidentiality.

© 2021 Song Z et al. *JAMA Health Forum.*

eTable 1. Race and Ethnic Composition of COVID-19 and Non-COVID-19 Hospitalizations in Medicare[1]

	Non-COVID-19 Patients		COVID-19 Patients
	Pre-COVID	During COVID	During COVID
Monthly hospitalizations	590,366	429,697	50,570
White (%)	81.2	81.5	73.3
Black (%)	11.7	11.5	15.1
Hispanic (%)	2.2	2.1	4.8
Remaining racial and ethnic minority groups (%)[2]	4.8	4.9	6.8

[1] Data are from the Centers for Medicare and Medicaid Services 100% inpatient claims files, which capture all Medicare fee-for-service hospitalizations. Pre-Covid denotes January 2019 through February 2020; during Covid denotes April 2020 through February 2021.
[2] Remaining racial and ethnic minority group members included Asian, North American Native, and beneficiaries of “unknown” or “other” race as reported by the Medicare beneficiary race variable from the Social Security Administration.

© 2021 Song Z et al. *JAMA Health Forum.*

eTable 2. Changes in Monthly Hospitalizations by Racial and Ethnic Group and Clinical Category[1]

	White			Black			Hispanic			Remaining Racial and Ethnic Minority Groups[2]		
	Pre-COVID	During COVID	% Change	Pre-COVID	During COVID	% Change	Pre-COVID	During COVID	% Change	Pre-COVID	During COVID	% Change
**COVID-19 Hospitalizations**	--	37.1	--	--	7.6	--	--	2.4	--	--	3.4	--
**Non-COVID-19 Hospitalizations**	479.6	350.2	-27.0	69.4	49.4	-28.7	13.2	8.9	-32.8	28.2	21.2	-24.8
Sepsis	40.8	29.6	-27.5	5.4	4.1	-24.8	1.4	0.9	-36.0	2.9	2.1	-27.5
Ventilators	3.1	2.1	-31.2	0.7	0.5	-27.8	0.1	0.1	-25.6	0.2	0.1	-28.3
Neurologic	39.6	32.5	-18.1	6.3	4.9	-22.6	1.1	0.8	-25.8	2.5	2.1	-16.4
Respiratory	57.5	30.4	-47.1	7.2	3.9	-45.6	1.3	0.6	-53.9	3.0	1.5	-47.7
Cardiac	96.9	73.2	-24.4	15.6	11.0	-29.1	2.5	1.7	-32.9	5.5	4.2	-23.9
Gastrointestinal	57.1	45.1	-21.1	7.9	5.9	-26.2	1.7	1.2	-29.7	3.5	2.9	-18.9
Orthopedic	76.9	50.2	-34.7	5.7	3.5	-38.1	1.1	0.7	-37.7	3.5	2.3	-35.0
Renal/genitourinary	37.6	29.2	-22.5	6.7	4.9	-26.3	1.3	0.9	-31.6	2.3	1.8	-21.3
Cancer	9.5	8.0	-16.0	2.0	1.6	-22.4	0.3	0.2	-26.5	0.7	0.6	-10.2
Other	60.6	49.9	-17.6	11.9	9.2	-22.9	2.4	1.8	-24.2	4.1	3.6	-13.4
**All Hospitalizations**	479.6	387.2	-19.3	69.4	57.1	-17.8	13.2	11.3	-14.3	28.2	24.6	-12.7

[1] Monthly hospitalizations are shown in 1,000s. Pre-COVID denotes January 2019 through February 2020; during COVID denotes April 2020 through February 2021. Percent (%) change is calculated relative to the pre-COVID baseline.
[2] Remaining racial and ethnic minority group members included Asian, North American Native, and beneficiaries of “unknown” or “other” race as reported by the Medicare beneficiary race variable from the Social Security Administration.

© 2021 Song Z et al. *JAMA Health Forum.*

eTable 3. Changes in Monthly Hospitalizations by Racial and Ethnic Group and Hospital Location[1]

	White			Black			Hispanic			Remaining Racial and Ethnic Minority Groups[2]		
	Pre-COVID	During COVID	% Change	Pre-COVID	During COVID	% Change	Pre-COVID	During COVID	% Change	Pre-COVID	During COVID	% Change
**COVID-19 Hospitalizations**	--	37.08	--	--	7.62	--	--	2.44	--	--	3.43	--
Urban hospitals	--	32.16	--	--	6.90	--	--	2.35	--	--	3.15	--
Rural hospitals	--	4.92	--	--	0.72	--	--	0.09	--	--	0.29	--
**Non-COVID-19 Hospitalizations**	479.56	350.16	-27.0	69.36	49.43	-28.7	13.23	8.90	-32.8	28.21	21.21	-24.8
Urban hospitals	428.88	314.65	-26.6	64.55	46.19	-28.5	12.87	8.65	-32.7	26.35	19.84	-24.7
Rural hospitals	50.68	35.51	-29.9	4.81	3.25	-32.6	0.37	0.24	-33.8	1.85	1.37	-26.3
**All Hospitalizations**	479.56	387.24	-19.3	69.37	57.05	-17.8	13.23	11.34	-14.3	28.21	24.64	-12.7
Urban hospitals	428.89	346.82	-19.1	64.55	53.09	-17.8	12.87	11.00	-14.5	26.35	22.99	-12.8
Rural hospitals	50.68	40.43	-20.2	4.81	3.96	-17.7	0.37	0.33	-9.2	1.85	1.65	-11.0

[1] Monthly hospitalizations are shown in 1,000s. Pre-COVID denotes January 2019 through February 2020; during COVID denotes April 2020 through February 2021. Percent (%) change is calculated relative to the pre-COVID baseline.
[2] Remaining racial and ethnic minority group members included Asian, North American Native, and beneficiaries of "unknown" or "other" race as reported by the Medicare beneficiary race variable from the Social Security Administration.

© 2021 Song Z et al. *JAMA Health Forum.*

eTable 4. In-Hospital Mortality, Black vs. White and Hispanic vs. White, Remaining Racial and Ethnic Minority Groups vs. White[1]

	White		Racial and Ethnic Minority Group		Between-group Difference[2]			
	Pre-COVID	During COVID	Pre-COVID	During COVID	Un-adjusted	Adjusted	95% CI	Change (%)
**Black vs. White**								
COVID-19 Hospitalizations	--	16.63	--	16.97	0.34	0.11	-0.24 to 0.47	--
Discharges to hospice included in mortality[3]	--	22.41	--	20.73	-1.68	-1.08	-1.48 to -0.69	--
Death during 30-day readmissions included in mortality[4]	--	19.03	--	19.26	0.23	0.33	-0.04 to 0.70	--
All-cause death within 30-days (a)[5]	--	27.79	--	25.58	-2.22	-0.89	-1.30 to -0.47	--
All-cause death within 30-days (b)[5]	--	27.82	--	25.62	-2.20	-0.89	-1.30 to -0.48	--
Admitted from skilled nursing facilities	--	7.42	--	8.25	0.83	0.43	0.11 to 0.75	--
Non-COVID-19 Hospitalizations	2.80	3.30	2.75	3.55	0.30	0.48	0.33 to 0.63	17.5
Discharges to hospice included in mortality	5.99	7.22	5.29	6.79	0.26	0.53	0.32 to 0.73	10.0
Death during 30-day readmissions included in mortality	3.55	4.24	3.61	4.73	0.43	0.56	0.39 to 0.73	15.5
All-cause death within 30-days (a)	8.15	9.72	7.11	9.05	0.37	0.56	0.31 to 0.80	7.9
All-cause death within 30-days (b)	8.30	9.89	7.25	9.21	0.37	0.59	0.34 to 0.84	8.1
**Hispanic vs. White**								
COVID-19 Hospitalizations	--	16.63	--	21.72	5.09	3.49	2.90 to 4.08	--
Discharges to hospice included in mortality	--	22.41	--	25.42	3.01	2.89	2.25 to 3.52	--
Death during 30-day readmissions included in mortality[3]	--	19.03	--	24.43	5.40	3.82	3.21 to 4.44	--
All-cause death within 30-days (a)	--	27.79	--	29.70	1.91	2.34	1.68 to 2.99	--
All-cause death within 30-days (b)	--	27.82	--	29.73	1.91	2.34	1.68 to 2.99	--
Admitted from skilled nursing facilities	--	7.42	--	3.86	-3.56	-3.74	-4.21 to -3.27	--
Non-COVID-19 Hospitalizations	2.80	3.30	2.77	3.58	0.30	0.29	-0.04 to 0.62	10.6
Discharges to hospice included in mortality	5.99	7.22	5.31	6.82	0.27	0.49	0.05 to 0.92	9.2
Death during 30-day readmissions included in mortality[3]	3.55	4.24	3.55	4.71	0.47	0.47	0.10 to 0.83	13.1
All-cause death within 30-days (a)	8.15	9.72	6.78	8.84	0.48	0.65	0.16 to 1.15	9.6
All-cause death within 30-days (b)	8.30	9.89	6.90	8.98	0.48	0.68	0.18 to 1.18	9.9
**Remaining Racial and Ethnic Minority Groups vs. White[6]**								
COVID-19 Hospitalizations	--	16.63	--	20.97	4.34	3.50	2.95 to 4.06	--
Discharges to hospice included in mortality	--	22.41	--	24.27	1.86	2.47	1.90 to 3.04	--
Death during 30-day readmissions included in mortality[3]	--	19.03	--	23.97	4.94	3.95	3.37 to 4.53	--
All-cause death within 30-days (a)	--	27.79	--	29.47	1.68	2.86	2.25 to 3.47	--
All-cause death within 30-days (b)	--	27.82	--	29.51	1.69	2.86	2.26 to 3.47	--
Admitted from skilled nursing facilities	--	7.42	--	6.15	-1.28	-2.31	-2.70 to -1.91	--
Non-COVID-19 Hospitalizations	2.80	3.30	3.32	3.79	-0.03	0.07	-0.17 to 0.30	2.4
Discharges to hospice included in mortality	5.99	7.22	5.79	7.01	-0.02	0.21	-0.11 to 0.54	3.5
Death during 30-day readmissions included in mortality[3]	3.55	4.24	4.23	4.92	0.01	0.03	-0.24 to 0.31	1.0
All-cause death within 30-days (a)	8.15	9.72	7.69	9.34	0.08	0.24	-0.13 to 0.62	3.0
All-cause death within 30-days (b)	8.30	9.89	7.87	9.54	0.09	0.29	-0.09 to 0.67	3.5

[1] Pre-COVID denotes January 2019 through February 2020; during COVID denotes April 2020 through February 2021.
[2] Between-group differences were cross-sectional differences for COVID-19 hospitalizations. For non-COVID-19 hospitalizations, between-group differences were differences in the changes between the racial/ethnic minority groups and White groups. Adjusted between-group differences were estimates from the statistical model, adjusted for age, sex, disability, end-stage renal disease (ESRD), month fixed effects, and hospital fixed effects, with standard errors clustered at the hospital level.
[3] In-hospital mortality reflects a discharge status of death. Among COVID-19 hospitalizations, a sensitivity analysis included discharges to hospice in the definition of in-hospital mortality.
[4] A sensitivity analysis included in-hospital deaths during 30-day readmissions to the index hospitalizations in the definition of mortality. Any hospitalization within 30 days of the index discharge date was considered a readmission, unless the subsequent hospitalization had a diagnosis of COVID-19 or any of the DRGs included on CMS's Bundled Payments for Care Improvement (BPCI) advanced MS-DRG exclusions list.
[5] A sensitivity analysis replaced in-hospital mortality with all-cause mortality within 30 days of the index hospitalization using beneficiary death date from the Medicare enrollment files. Definition (a) attributed death to the index hospitalization when an additional hospitalization within 30 days occurred for COVID-19 or an advanced excluded MS-DRG (i.e., a new index hospitalization and not a readmission); definition (b) attributed death to the first index hospitalization and the additional index hospitalization when an additional hospitalization within 30 days occurred for COVID-19 or an advanced excluded MS-DRG (i.e., a new index hospitalization and not a readmission).

© 2021 Song Z et al. *JAMA Health Forum.*

[6] Remaining racial and ethnic minority group members included Asian, North American Native, and beneficiaries of “unknown” or “other” race as reported by the Medicare beneficiary race variable from the Social Security Administration.

© 2021 Song Z et al. *JAMA Health Forum.*

eTable 5. Differential Changes in Hospitalization Outcomes[1]

	**Black vs. White**		**Hispanic vs. White**		**Remaining Racial and Ethnic Minority Groups vs. White[2]**	
	Adjusted estimate	95% CI	Adjusted estimate	95% CI	Adjusted estimate	95% CI
**COVID-19 Hospitalizations**						
In-hospital mortality[3]	0.11	-0.24 to 0.47	3.49	2.90 to 4.08	3.50	2.95 to 4.06
Urban Hospitals	0.08	-1.05 to 1.20	3.11	0.93 to 5.29	4.47	2.86 to 6.08
Rural Hospitals	0.12	-0.26 to 0.49	3.48	2.88 to 4.09	3.42	2.84 to 4.00
Discharge to hospice	-1.20	-1.40 to -1.00	-0.60	-0.91 to -0.29	-1.03	-1.29 to -0.78
Urban Hospitals	-0.90	-1.44 to -0.36	0.60	-0.46 to 1.66	-0.56	-1.25 to 0.13
Rural Hospitals	-1.22	-1.43 to -1.00	-0.63	-0.95 to -0.30	-1.05	-1.32 to -0.78
Discharge to post-acute care	1.56	1.13 to 1.99	-9.34	-9.91 to -8.77	-5.76	-6.25 to -5.26
Urban Hospitals	3.06	1.96 to 4.16	-7.86	-9.92 to -5.79	-2.33	-4.02 to -0.64
Rural Hospitals	1.41	0.95 to 1.87	-9.40	-9.99 to -8.82	-5.98	-6.50 to -5.47
**Non-COVID-19 Hospitalizations**						
In-hospital mortality	0.48	0.33 to 0.63	0.29	-0.04 to 0.62	0.07	-0.17 to 0.30
Urban Hospitals	0.59	0.10 to 1.08	0.35	-1.24 to 1.94	-0.53	-1.35 to 0.29
Rural Hospitals	0.48	0.32 to 0.63	0.30	-0.04 to 0.63	0.11	-0.13 to 0.36
Discharge to hospice	0.05	-0.09 to 0.19	0.19	-0.11 to 0.50	0.14	-0.07 to 0.36
Urban Hospitals	0.18	-0.40 to 0.76	-1.49	-3.60 to 0.62	0.32	-0.45 to 1.09
Rural Hospitals	0.03	-0.11 to 0.18	0.23	-0.08 to 0.54	0.13	-0.10 to 0.35
Discharge to post-acute care	0.29	-0.07 to 0.66	0.32	-0.41 to 1.05	0.47	-0.02 to 0.96
Urban Hospitals	0.67	-0.65 to 1.98	1.94	-2.53 to 6.41	-0.01	-1.67 to 1.65
Rural Hospitals	0.29	-0.09 to 0.67	0.32	-0.43 to 1.06	0.52	0.01 to 1.04

[1] Between-group differences were changes in outcomes during the COVID-19 pandemic (April 2020-February 2021) relative to pre-COVID-19 (January 2019-February 2020) in the racial/ethnic minority groups relative to those changes in the comparison (White) group, estimated using the main specification of the statistical model with a difference-in-differences approach adjusted for age, sex, disability, end-stage renal disease (ESRD), month fixed effects, and hospital fixed effects, with standard errors clustered at the hospital level.
[2] Remaining racial and ethnic minority group members included Asian, North American Native, and beneficiaries of "unknown" or "other" race as reported by the Medicare beneficiary race variable from the Social Security Administration.
[3] In-hospital mortality denotes a discharge status of death.

© 2021 Song Z et al. *JAMA Health Forum.*

eTable 6. Characteristics of Medicare Hospitalizations by Discharge Status

A. Hospitalizations with a discharge status of death (in-hospital death)

	White			Black			Hispanic			Remaining Racial and Ethnic Minority Groups[1]		
	Pre-COVID	During COVID		Pre-COVID	During COVID		Pre-COVID	During COVID		Pre-COVID	During COVID	
		Non-COVID	COVID		Non-COVID	COVID		Non-COVID	COVID		Non-COVID	COVID
Hospitalizations (no.)[2]	187,816	127,284	67,833	26,666	19,316	14,221	5,129	3,500	5,831	13,101	8,851	7,911
Share of total (%)	2.8	3.3	16.6	2.7	3.6	17.0	2.8	3.6	21.7	3.3	3.8	21.0
Age (yrs)	77.7	77.2	78.2	72.5	72.1	74.3	74.0	72.6	73.4	75.5	74.9	75.9
Sex (% Female)	49.1	48.3	42.9	51.9	51.8	49.2	48.3	47.5	39.2	45.3	44.3	41.0
Risk score[3]	3.14	3.16	2.59	3.39	3.36	2.72	3.21	3.23	2.43	3.30	3.32	2.51
ESRD (%)[4]	4.9	5.0	3.5	19.3	20.0	13.3	18.9	20.3	13.7	12.9	13.2	9.6
Disability (%)	8.6	8.7	5.8	19.1	19.0	14.2	19.9	22.5	15.8	10.7	10.5	7.9
Dual eligible (%)	19.7	19.6	27.5	44.8	45.9	55.1	44.6	46.5	43.2	30.1	30.5	36.3

B. Hospitalizations with a discharge to hospice

	White			Black			Hispanic			Remaining Racial and Ethnic Minority Groups[1]		
	Pre-COVID	During COVID		Pre-COVID	During COVID		Pre-COVID	During COVID		Pre-COVID	During COVID	
		Non-COVID	COVID		Non-COVID	COVID		Non-COVID	COVID		Non-COVID	COVID
Hospitalizations (no.)[2]	214,037	150,754	23,560	24,727	17,595	3,150	4,714	3,171	992	9,767	7,492	1,247
Share of total (%)	3.2	3.9	5.8	2.5	3.2	3.8	2.5	3.2	3.7	2.5	3.2	3.3
Age (yrs)	80.9	80.6	82.7	77.2	77.2	80.2	80.6	79.3	81.3	78.6	78.3	81.0
Sex (% Female)	55.2	54.5	54.1	57.6	58.6	57.3	56.3	54.9	52.7	50.9	51.5	49.6
Risk score[3]	3.17	3.23	2.60	3.45	3.48	2.84	3.22	3.28	2.65	3.35	3.42	2.66
ESRD (%)[4]	2.2	2.2	1.8	7.2	7.2	6.6	5.7	7.4	5.3	4.8	5.2	5.4
Disability (%)	5.2	5.0	3.1	11.1	10.4	5.8	10.0	11.7	7.1	6.8	6.1	3.7
Dual eligible (%)	20.0	18.5	30.9	46.6	46.4	65.2	64.4	62.8	67.9	38.7	37.0	49.1

© 2021 Song Z et al. *JAMA Health Forum.*

C. Hospitalizations with a discharge to post-acute care

	White			Black			Hispanic			Remaining Racial and Ethnic Minority Groups[1]		
	Pre-COVID	During COVID		Pre-COVID	During COVID		Pre-COVID	During COVID		Pre-COVID	During COVID	
		Non-COVID	COVID		Non-COVID	COVID		Non-COVID	COVID		Non-COVID	COVID
Hospitalizations (no.)[2]	1,618,444	788,965	93,582	212,702	102,721	20,281	33,259	14,163	3,884	76,761	36,084	6,318
Share of total (%)	24.1	20.5	22.9	21.9	18.9	24.2	18.0	14.5	14.5	19.4	15.5	16.7
Age (yrs)	78.6	78.2	78.6	73.0	72.3	73.6	74.1	72.3	74.2	76.0	74.9	76.1
Sex (% Female)	60.0	59.2	55.6	56.2	53.2	51.9	53.3	49.6	48.5	52.5	50.2	48.8
Risk score[3]	2.11	2.16	2.21	2.32	2.42	2.34	2.22	2.33	2.22	2.15	2.23	2.21
ESRD (%)[4]	3.2	3.2	3.7	14.0	14.3	14.5	13.3	14.3	14.4	8.5	8.9	9.4
Disability (%)	7.2	7.4	7.7	17.4	18.4	15.2	20.0	22.8	17.4	8.9	9.8	9.2
Dual eligible (%)	26.1	26.7	42.4	56.1	58.9	70.6	78.5	78.3	84.0	51.0	48.7	65.2

[1] Remaining racial and ethnic minority group members included Asian, North American Native, and beneficiaries of "unknown" or "other" race as reported by the Medicare beneficiary race variable from the Social Security Administration.
[2] Total number of hospitalizations in Medicare fee-for-service.
[3] The risk score was calculated using the Centers for Medicare and Medicaid Services Hierarchical Condition Category (CMS-HCC) model with the beneficiaries' age, sex, and inpatient clinical diagnoses.
[4] ESRD denotes end stage renal disease.

© 2021 Song Z et al. *JAMA Health Forum.*

eTable 7. Absent Non-COVID-19 Inpatients During the Pandemic

	White			Black			Hispanic			Remaining Racial and Ethnic Minority Groups[1]		
Non-COVID-19 Patients	Pre-COVID	During COVID	Absent During COVID	Pre-COVID	During COVID	Absent During COVID	Pre-COVID	During COVID	Absent During COVID	Pre-COVID	During COVID	Absent During COVID
Hospitalizations	141,674	103,142	38,532	89,950	65,109	24,841	16,888	11,584	5,304	589,920	432,755	157,165
Age (yrs)	65.9	65.2	67.6	65.1	64.6	66.2	63.6	62.2	66.7	72.8	72.6	73.5
Sex (% female)	52.6	51.2	56.5	54.9	53.5	58.8	51.3	49.5	55.3	53.6	52.8	55.8
Risk score[2]	2.03	2.10	1.85	2.08	2.16	1.89	1.95	2.03	1.79	1.94	2.00	1.79
ESRD (%)[3]	18.0	18.9	15.5	19.5	20.6	16.6	21.2	22.7	17.9	4.3	4.4	4.0
Disability (%)	27.7	27.8	27.4	31.0	30.8	31.7	33.7	35.8	29.0	13.1	13.0	13.5
Dual eligible (%)	53.2	52.6	54.5	53.1	53.5	51.9	72.5	71.3	75.0	22.3	21.8	23.6

[1] Remaining racial and ethnic minority group members included Asian, North American Native, and beneficiaries of "unknown" or "other" race as reported by the Medicare beneficiary race variable from the Social Security Administration.
[2] The risk score was calculated using the Centers for Medicare and Medicaid Services Hierarchical Condition Category (CMS-HCC) model with the beneficiaries' age, sex, and inpatient clinical diagnoses.
[3] ESRD denotes end stage renal disease.

© 2021 Song Z et al. *JAMA Health Forum.*

eTable 8. Differential Changes in Risk Score[1]

$$risk_{iht} = \beta_0 + \sum_{t=1/19}^{t=3/20} \beta_t\left(R_i \times 1(month_t)\right) + \beta_{post}(R_i \times post_t) + \Gamma X_i + \theta_h + \delta_t + \varepsilon_{iht}$$

Non-COVID-19 Hospitalizations	**Black vs. White**		**Hispanic vs. White**		**Remaining Racial and Ethnic Minority Groups vs. White[2]**	
	Coeff (S.E.)	95% CI	Coeff (S.E.)	95% CI	Coeff (S.E.)	95% CI
Differential Change (β_{post})	0.024 (0.005)	0.015 to 0.034	0.015 (0.011)	0.015 to 0.034	0.021 (0.008)	0.006 to 0.036

[1] The Centers for Medicare and Medicaid Services Hierarchical Condition Category (CMS-HCC) risk score is a measure of expected spending typically reflecting the clinical burden of disease used by Medicare in risk adjustment and was calculated using the model beneficiaries' age, sex, and inpatient clinical diagnoses. This analysis examined changes in the risk score associated with racial and ethnic groups among non-COVID-19 hospitalizations.
[2] Remaining racial and ethnic minority group members included Asian, North American Native, and beneficiaries of "unknown" or "other" race as reported by the Medicare beneficiary race variable from the Social Security Administration.

© 2021 Song Z et al. *JAMA Health Forum.*

eTable 9. Sensitivity Analyses and Falsification Test, Black vs. White

	Black vs. White Between-group Differences[1]													
	Main Specification		Alternative Specification (1)		Alternative Specification (2)		Alternative Specification (3)		Alternative Specification (4)		Alternative Sample Age 65+ only		Falsification Specification	
	Coefficient	P value	Coefficient	P value	Coefficient	P value	Coefficient	P value	Coefficient	P value	Coefficient	P value	Coefficient	P value
**COVID-19 Hospitalizations[2]**														
In-hospital mortality[3]	0.11	0.53	0.24	0.19	0.75	< 0.001	0.09	0.63	0.21	0.23	0.33	0.12	--	--
Discharge to hospice	-1.20	< 0.001	-1.24	< 0.001	-1.05	< 0.001	-1.20	< 0.001	-1.10	< 0.001	-1.18	< 0.001	--	--
Discharge to post-acute care	1.56	< 0.001	1.87	< 0.001	1.22	< 0.001	1.57	< 0.001	1.43	< 0.001	2.62	< 0.001	--	--
**Non-COVID-19 Hospitalizations**														
In-hospital mortality	0.48	< 0.001	0.48	< 0.001	0.46	< 0.001	0.48	< 0.001	0.48	< 0.001	0.59	< 0.001	-0.19	0.05
Discharge to hospice	0.05	0.50	0.05	0.51	0.08	0.27	0.05	0.50	0.05	0.45	0.24	0.02	-0.03	0.77
Discharge to post-acute care	0.29	0.11	0.29	0.12	0.24	0.19	0.29	0.11	0.30	0.11	-0.57	0.02	0.38	0.10
**All Hospitalizations**														
In-hospital mortality	0.95	< 0.001	0.94	< 0.001	0.94	< 0.001	0.94	< 0.001	0.95	< 0.001	1.40	< 0.001	-0.18	0.06
Controls														
Age, sex	Yes		Yes		Yes		Yes		Yes		Yes		Yes	
Share ESRD[4], disability	Yes		No		Yes		Yes		Yes		Yes		Yes	
CBSA-level COVID-19 infection rate	No		No		No		Yes		No		No		No	
Hospital fixed effects	Yes		Yes		No		Yes		No		Yes		Yes	
Hospital random effects	No		No		No		No		Yes		No		No	

[1] Between-group differences were changes in outcomes during the COVID-19 pandemic (April 2020-February 2021) relative to pre-COVID-19 (January 2019-February 2020) in Black patients relative to those changes in the comparison (White) group, estimated using our statistical models shown here, with standard errors clustered at the hospital level. The alternative specifications (1) through (4) differ in their inclusion of controls, as shown in the bottom portion of the table. The alternative sample examined only aged beneficiaries who were 65 years or older. The falsification test estimated the main specification of the statistical model, using January-December 2019 as pre-COVID months and January-February 2020 as the COVID period.
[2] Given there were no COVID-19 hospitalizations pre-COVID, the between-group difference was a cross-sectional comparison between Black beneficiaries and White beneficiaries using data during the pandemic.
[3] In-hospital mortality denotes a discharge status of death.
[4] The risk score was calculated using the Centers for Medicare and Medicaid Services Hierarchical Condition Category (CMS-HCC) model with the beneficiaries' age, sex, and inpatient clinical diagnoses.
[5] ESRD denotes end stage renal disease.

© 2021 Song Z et al. *JAMA Health Forum.*

eTable 10. Sensitivity Analyses and Falsification Test, Hispanic vs. White

	Hispanic vs. White Between-group Differences[1]													
	**Main Specification**		**Alternative Specification (1)**		**Alternative Specification (2)**		**Alternative Specification (3)**		**Alternative Specification (4)**		**Alternative Sample Age 65+ only**		**Falsification Specification**	
	Coefficient	P value	Coefficient	P value	Coefficient	P value	Coefficient	P value	Coefficient	P value	Coefficient	P value	Coefficient	P value
**COVID-19 Hospitalizations[2]**														
In-hospital mortality[3]	3.49	< 0.001	3.61	< 0.001	6.08	< 0.001	3.47	< 0.001	4.31	< 0.001	4.20	< 0.001	--	--
Discharge to hospice	-0.60	< 0.001	-0.68	< 0.001	-0.96	< 0.001	-0.60	< 0.001	-0.88	< 0.001	-0.58	0.003	--	--
Discharge to post-acute care	-9.34	< 0.001	-9.05	< 0.001	-8.06	< 0.001	-9.34	< 0.001	-8.99	< 0.001	-9.18	< 0.001	--	--
**Non-COVID-19 Hospitalizations**														
In-hospital mortality	0.29	0.08	0.29	0.08	0.27	0.11	0.27	0.11	0.28	0.09	0.28	0.23	0.28	0.19
Discharge to hospice	0.19	0.21	0.19	0.22	0.22	0.16	0.19	0.23	0.20	0.21	0.54	0.03	-0.17	0.43
Discharge to post-acute care	0.32	0.39	0.33	0.38	0.01	0.97	0.34	0.37	0.32	0.40	-0.24	0.65	0.67	0.17
**All Hospitalizations**														
In-hospital mortality	2.86	< 0.001	2.86	< 0.001	2.83	< 0.001	2.62	< 0.001	2.85	< 0.001	3.99	< 0.001	0.28	0.19
Controls														
Age, sex	Yes		Yes		Yes		Yes		Yes		Yes		Yes	
Share ESRD[4], disability	Yes		No		Yes		Yes		Yes		Yes		Yes	
CBSA-level COVID-19 infection rate	No		No		No		Yes		No		No		No	
Hospital fixed effects	Yes		Yes		No		Yes		No		Yes		Yes	
Hospital random effects	No		No		No		No		Yes		No		No	

[1] Between-group differences were changes in outcomes during the COVID-19 pandemic (April 2020-February 2021) relative to pre-COVID-19 (January 2019-February 2020) in Hispanic patients relative to those changes in the comparison (White) group, estimated using our statistical models shown here, with standard errors clustered at the hospital level. The alternative specifications (1) through (4) differ in their inclusion of controls, as shown in the bottom portion of the table. The alternative sample examined only aged beneficiaries who were 65 years or older. The falsification test estimated the main specification of the statistical model, using January-December 2019 as pre-COVID months and January-February 2020 as the COVID period.
[2] Given there were no COVID-19 hospitalizations pre-COVID, the between-group difference was a cross-sectional comparison between Hispanic beneficiaries and White beneficiaries using data during the pandemic.
[3] In-hospital mortality denotes a discharge status of death.
[4] The risk score was calculated using the Centers for Medicare and Medicaid Services Hierarchical Condition Category (CMS-HCC) model with the beneficiaries' age, sex, and inpatient clinical diagnoses.
[5] ESRD denotes end stage renal disease.

© 2021 Song Z et al. *JAMA Health Forum.*

eTable 11. Sensitivity Analyses and Falsification Test, Remaining Racial and Ethnic Minority Groups vs. White

	Remaining Racial and Ethnic Minority Groups vs. White[1] Between-group Differences[2]													
	**Main Specification**		**Alternative Specification (1)**		**Alternative Specification (2)**		**Alternative Specification (3)**		**Alternative Specification (4)**		**Alternative Sample Age 65+ only**		**Falsification Specification**	
	Coefficient	P value	Coefficient	P value	Coefficient	P value	Coefficient	P value	Coefficient	P value	Coefficient	P value	Coefficient	P value
**COVID-19 Hospitalizations[3]**														
In-hospital mortality[4]	3.49	< 0.001	3.61	< 0.001	6.08	< 0.001	3.47	< 0.001	4.31	< 0.001	4.20	< 0.001	--	--
Discharge to hospice	-0.60	< 0.001	-0.68	< 0.001	-0.96	< 0.001	-0.60	< 0.001	-0.88	< 0.001	-0.58	0.003	--	--
Discharge to post-acute care	-9.34	< 0.001	-9.05	< 0.001	-8.06	< 0.001	-9.34	< 0.001	-8.99	< 0.001	-9.18	< 0.001	--	--
**Non-COVID-19 Hospitalizations**														
In-hospital mortality	0.29	0.08	0.29	0.08	0.27	0.11	0.27	0.11	0.28	0.09	0.28	0.23	0.28	0.19
Discharge to hospice	0.19	0.21	0.19	0.22	0.22	0.16	0.19	0.23	0.20	0.21	0.54	0.03	-0.17	0.43
Discharge to post-acute care	0.32	0.39	0.33	0.38	0.01	0.97	0.34	0.37	0.32	0.40	-0.24	0.65	0.67	0.17
**All Hospitalizations**														
In-hospital mortality	2.86	< 0.001	2.86	< 0.001	2.83	< 0.001	2.62	< 0.001	2.85	< 0.001	3.99	< 0.001	0.28	0.19
Controls														
Age, sex	Yes		Yes		Yes		Yes		Yes		Yes		Yes	
Share ESRD[4], disability	Yes		No		Yes		Yes		Yes		Yes		Yes	
CBSA-level COVID-19 infection rate	No		No		No		Yes		No		No		No	
Hospital fixed effects	Yes		Yes		No		Yes		No		Yes		Yes	
Hospital random effects	No		No		No		No		Yes		No		No	

[1] Remaining racial and ethnic minority group members included Asian, North American Native, and beneficiaries of "unknown" or "other" race as reported by the Medicare beneficiary race variable from the Social Security Administration.
[2] Between-group differences were changes in outcomes during the COVID-19 pandemic (April 2020-February 2021) relative to pre-COVID-19 (January 2019-February 2020) in the racial and ethnic minority groups relative to those changes in the comparison (White) group, estimated using our statistical models shown here, with standard errors clustered at the hospital level. The alternative specifications (1) through (4) differ in their inclusion of controls, as shown in the bottom portion of the table. The alternative sample examined only aged beneficiaries who were 65 years or older. The falsification test estimated the main specification of the statistical model, using January-December 2019 as pre-COVID months and January-February 2020 as the COVID period.
[3] Given there were no COVID-19 hospitalizations pre-COVID, the between-group difference was a cross-sectional comparison between beneficiaries of the racial and ethnic minority groups and White beneficiaries using data during the pandemic.
[3] In-hospital mortality denotes a discharge status of death.
[4] The risk score was calculated using the Centers for Medicare and Medicaid Services Hierarchical Condition Category (CMS-HCC) model with the beneficiaries' age, sex, and inpatient clinical diagnoses.
[5] ESRD denotes end stage renal disease.

© 2021 Song Z et al. *JAMA Health Forum.*
